# Small Organic Molecule Based on Benzothiadiazole for Electrocatalytic Hydrogen Production

**DOI:** 10.1021/jacs.1c10600

**Published:** 2021-12-02

**Authors:** Martin Axelsson, Cleber F. N. Marchiori, Ping Huang, C. Moyses Araujo, Haining Tian

**Affiliations:** † Department of Chemistry-Ångström Laboratory, Uppsala University, Uppsala SE 751 20, Sweden; ‡ Department of Engineering and Physics, Karlstad University, Karlstad 65188, Sweden; § Department of Physics and Astronomy, Ångström Laboratory, Uppsala University, Uppsala 751 20, Sweden

## Abstract

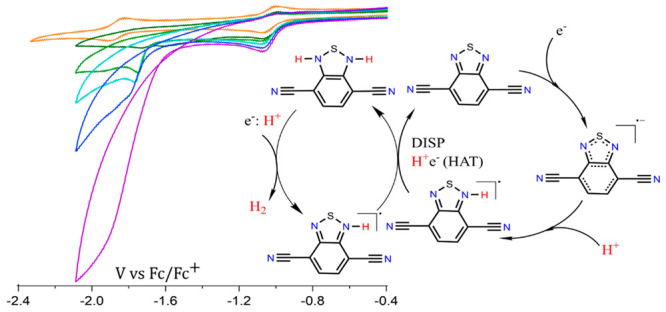

A small organic molecule 2,1,3-benzothiadiazole-4, 7-dicarbonitrile (BTDN) is assessed for electrocatalytic hydrogen evolution on glassy carbon electrode and shows a hydrogen production Faradaic efficiency of 82% in the presence of salicylic acid. The key catalytic intermediates of reduced species BTDN^–•^ and protonated intermediates are characterized or hypothesized by using various spectroscopic methods and density functional theory (DFT)-based calculations. With the experimental and theoretical results, a catalytic mechanism of BTDN for electrocatalytic H_2_ evolution is proposed.

Using photocatalysis and electrocatalysis to generate clean and renewable fuels from abundant resources such as water and CO_2_ is one of the most promising directions for replacing fossil fuels.^1^ Development of low-cost, efficient, and environmentally friendly catalysts is therefore vital. To date, the catalysts for these processes have commonly been metal complexes^2,3^ and metallic/organic materials.^4,5^ While often being utilized as ligands in metal complexes or building blocks in organic materials, small organic molecules have not been well studied as catalysts for these types of redox reactions. Recently, small, aromatic, and nitrogen-rich organic compounds have been demonstrated to facilitate both water oxidation^6,7^ and CO_2_ reduction.^8−12^ When it comes to hydrogen evolution, there are very few reported cases that we know of.^13^ 2,1,3-benzothiadiazole (BT) has been used as a popular electron acceptor blocking unit in many organic polymers for photocatalysis, and we have previously proposed that BT could be an active site for hydrogen production in polymer dots photocatalyst.^14−16^ In this work, we experimentally and theoretically assessed a BT derivative 2,1,3-benzothiadiazole-4, 7-dicarbonitrile (BTDN, see Scheme 1) for electrocatalytic hydrogen production and investigated its catalytic mechanism.

**Scheme 1:**
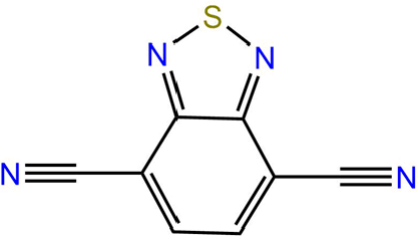
Molecular Structure of 2,1,3-Benzothiadiazole-4, 7-dicarbonitrile (BTDN)

Nitrile (CN) groups at the 4 and 7 positions of BTDN have characteristic absorption in the infrared (IR) region, which is vital for monitoring reaction intermediates from the IR spectrum in the following study. Inspired by the study from Cole-Hamilton and coauthors on the photochemical properties of BTDN in micelles,^17−19^ we studied BTDN’s electrocatalytic properties in the work. The electrochemical behavior of BTDN is evaluated by cyclic voltammetry (CV), rendering two fully reversible redox waves at −1.06 and −1.88 V vs ferrocene/ferrocenium (Fc/Fc^+^) in acetonitrile, as shown in Figure 1a. The two redox events represent the reduction of BTDN to its anionic radical species BTDN^–•^ and doubly reduced species BTDN^2–^, respectively. To check if the reduced species of BTDN can interact with proton, we selected salicylic acid (SAL) with a p*K*_a_ of 16.7 in acetonitrile (AcN)^20^ as the organic acid for this experiment after comparing it with trifluoroacetic acid and acetic acid (Figure S1). SAL offers a broad electrochemical window that allows us to observe the catalytic behavior of BTDN in the presence of protons (Figure S2).

**Figure 1:**
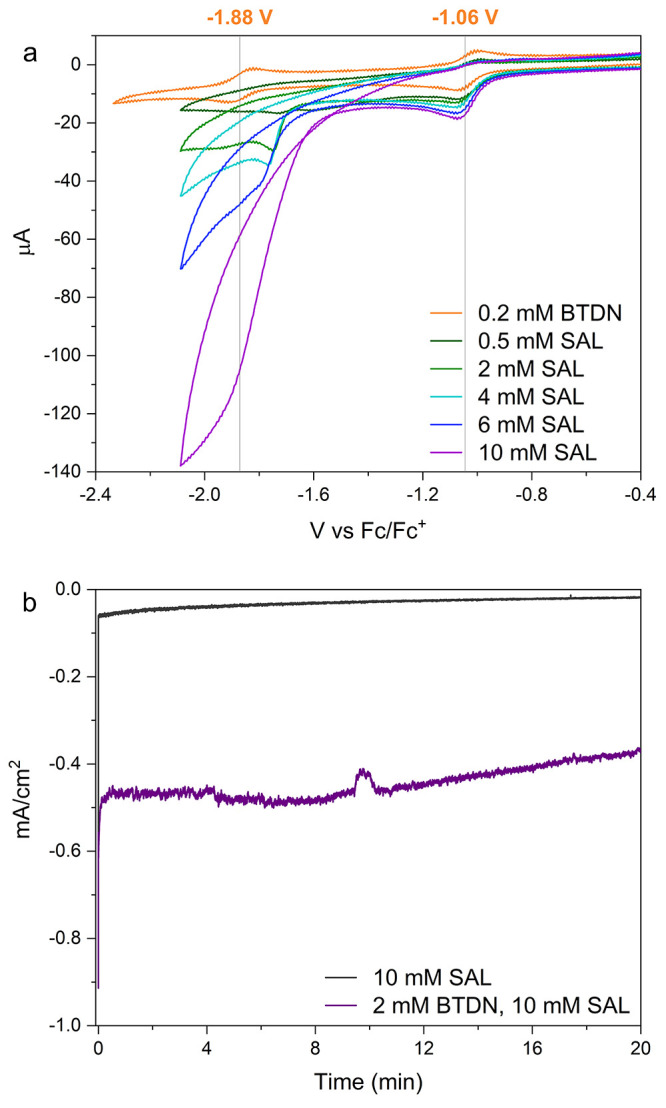
(a) Cyclic voltammetry of BTDN combined with titration of SAL, going from two reversible redox waves for only BTDN (orange) to the fully catalytic behavior with 50 equiv. of SAL (purple). (b) Current from electrocatalytic H_2_ generation at −1.65 V vs Fc/Fc^+^ from 10 mM SAL (black) and 2 mM BTDN with 10 mM SAL (purple).

When SAL was introduced to the system as the proton donor, the CV of BTDN (Figure 1a) was significantly perturbed. One can see that the first reduction peak shifts to a less negative potential as compared to that of the pure BTDN as shown in Figure 1a, which is characteristic of an EC (an electrochemical step E, followed by a chemical step C) type process.^21^ At low concentrations, around 50 μM BTDN, the shift in the voltage peak with respect to the scan rate and the concentration of the acid correlates well with the prediction of an EC mechanism (Figures S10 and 11).^22^ This can be attributed to a single protonation of the BTDN^–•^ species forming an organo-hydride species BTDNH^•^. At higher concentrations, around 250 μM, increased reductive current is identified when the concentration of acid is increased (Figure 1). It suggests this reaction probably is a homogeneous electron transfer similar to a disproportionation mechanism (DISP), which is further supported by simulations (Figure S12). The electron transfer is coupled to a proton transfer that gives a net hydrogen atom transfer (HAT), probably to form a new doubly protonated intermediate, which will be discussed later on with the help from IR data and DFT calculations. Notably, at the higher concentrations, the second redox peak completely disappears and gives way for a catalytic wave with the foot of the wave starting around −1.6 V vs Fc/Fc^+.^

A titration of BTDN with SAL in CVs measured at every step was subsequently carried out. The corresponding data are shown in Figure 1a. The concentration of SAL was used from 2.5 to 50 equiv. After passing two equivalents of acid, all oxidative current from the second reduction disappears, meaning that all the reduced species have reacted at this point.

To confirm that the catalytic wave appearing is in fact hydrogen evolution, we conducted bulk electrolysis experiments. The electrolysis was performed in the foot of the catalytic wave at −1.65 V vs Fc/Fc^+^ to avoid a large hydrogen evolution from bare SAL and glassy carbon electrode. In the presence of BTDN (Figure 1b), a current density of 0.5 mA cm^–2^ was achieved and only 20% decrease in current in 20 min was observed. Hydrogen evolution was also detected by gas chromatography (Figure S9) and a Faradaic efficiency was determined to be 82%, the loss can be attributed to the degradation of SAL on the surface of the electrode or decomposition of some BTDN. Without BTDN, only a 0.06 mA cm^–2^ current was obtained and decreased rapidly. Only a tiny amount of hydrogen was produced with a very low Faradaic efficiency of 25%. To appraise the TON of the catalyst, we ran a longer experiment in a 3 mL solution with a 0.2 mM BTDN catalyst (Figure S13), which evolved into 8.1 μmol of H_2_, giving a TON of 13. This result suggests that the BTDN indeed works for electrocatalytic hydrogen production. It is worth noting that TON is counted with all BTDN in the reactor, not only the BTDN participating in catalysis on the electrode.

To investigate the catalytic mechanism of BTDN for proton reduction, we attempted to monitor the reaction intermediates by electron paramagnetic resonance (EPR), spectroelectrochemistry (SEC), FTIR spectroscopy and nuclear magnetic resonance (NMR). Because of the reduction occurs before any chemical step in the first reduction peak, the first intermediate is determined to be the BTDN^–•^ radical anion. The radical is also previously mentioned in literature and is incredibly stable.^17^ The BTDN^–•^ radical was therefore generated through bulk electrolysis in an inert environment, showing NMR silent in aromatic region (Figure S14). EPR measurement of the BTDN^–•^ (Figure S15) gave 27 splitting peaks. It means that the radical has hyperfine coupling to two N atom site. This result is in consistent with previously reported data and indicates that the radical is completely delocalized over the entire molecule,^17^ which is also consistent with our DFT calculations (Figure S20).

To further probe the intermediates during the catalytic cycle, we performed SEC in an inert atmosphere. First, BTDN was studied in the absence of the organic acid. The result is shown in Figure 2a and Figure S16. One can see the characteristic absorption from BTDN^–•^ (Figure S15) appears when the applied potential (−1.1 V vs Fc/Fc^+^) reaches the first redox potential of BTDN. When a more negative potential (−1.9 V vs Fc/Fc^+^) is applied, the doubly reduced species BTDN^2–^ appeared with new absorption bands at 520 and 555 nm (). Subsequently, the SEC experiment in the presence of 10 equiv. of SAL was carried out, as shown in Figure 2b. Notably, all features from BTDN^–•^ and BTDN^2–^ completely disappear. Instead, there is a new species with an absorption feature at 340 nm appearing at a potential of −1.1 V vs Fc/Fc^+^, which is attributed to the protonation of BTDN^–•^. When a catalytic potential of −1.9 V vs Fc/Fc^+^ is applied, the feature completely vanishes, which could be attributed to the reformation of the BTDN after catalysis. The bleach at 320 nm could be from decomposition of SAL, which absorbs in this region,^23^ and from decomposition of BTDN.

**Figure 2:**
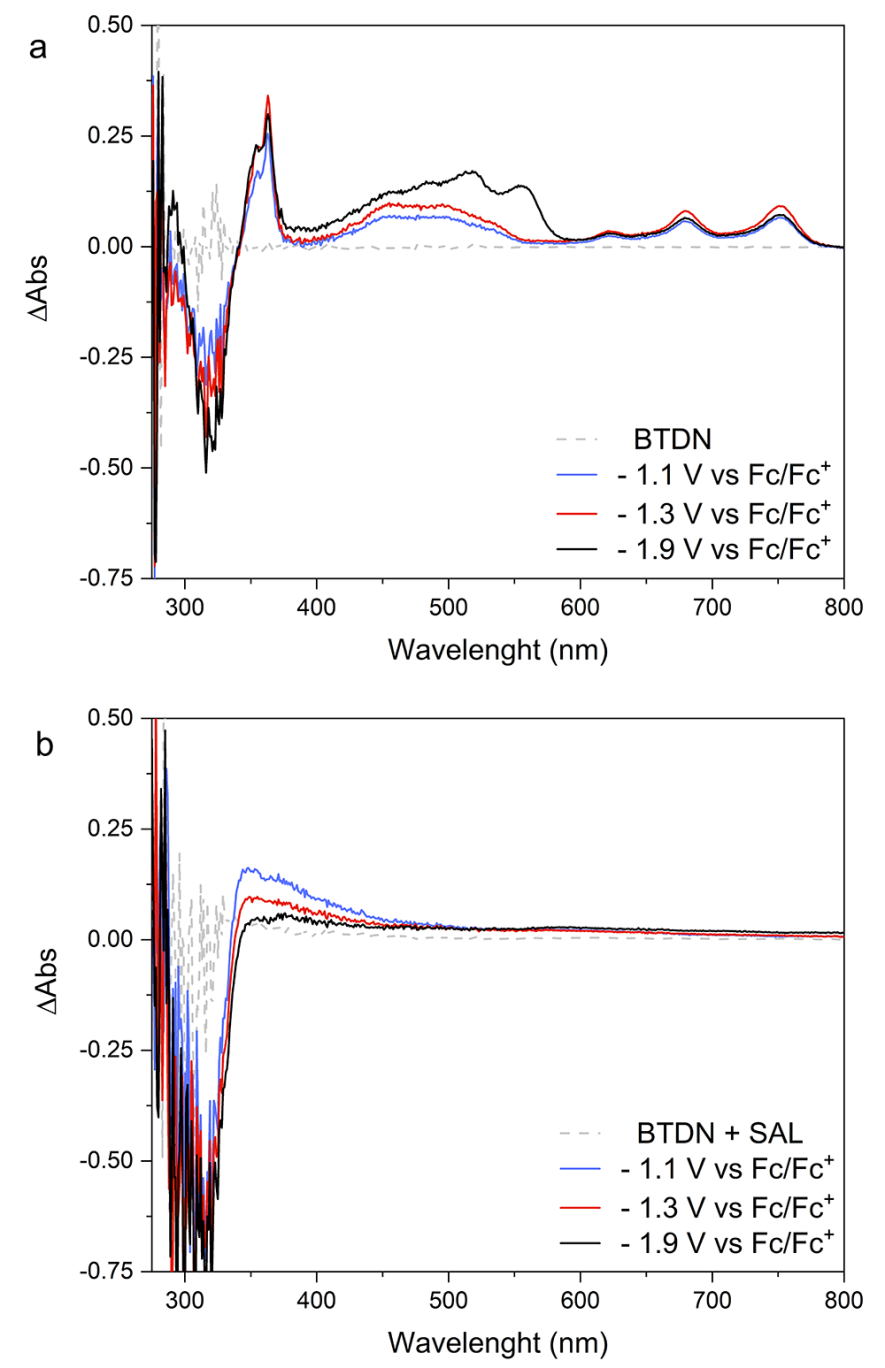
Differential spectra from UV–vis SEC of (a) BTDN and (b) BTDN with SAL at three different potentials of −1.1 V (blue), −1.3 V (red), and −1.9 V vs Fc/Fc^+^ (black).

FTIR was used to characterize different intermediates generated from the bulk electrolysis. The corresponding data are shown in Figure 3. To follow the changes of the molecule, the characteristic absorption from C≡N bond stretching is used as an IR probe.^24^ the stretching mode of BTDN absorbs quite weakly as a singular peak at 2235 cm^–1^. When BTDN was reduced to BTDN^–•^, the absorption peak shifted to a lower value of 2184 cm^–1^. Such a shift resulted from the delocalized nature of the radical, making the nitrile bond as a part of the conjugated system in BTDN. This also leads to that intensity of the nitrile stretching absorption becomes much stronger, about 10 times as compared to that of the BTDN. When 1 equiv. of SAL was added to BTDN^–•^ solution, the 2184 cm^–1^ peak started to disappear; meanwhile, two new peaks at 2235 and 2217 cm^–1^ appeared.

**Figure 3:**
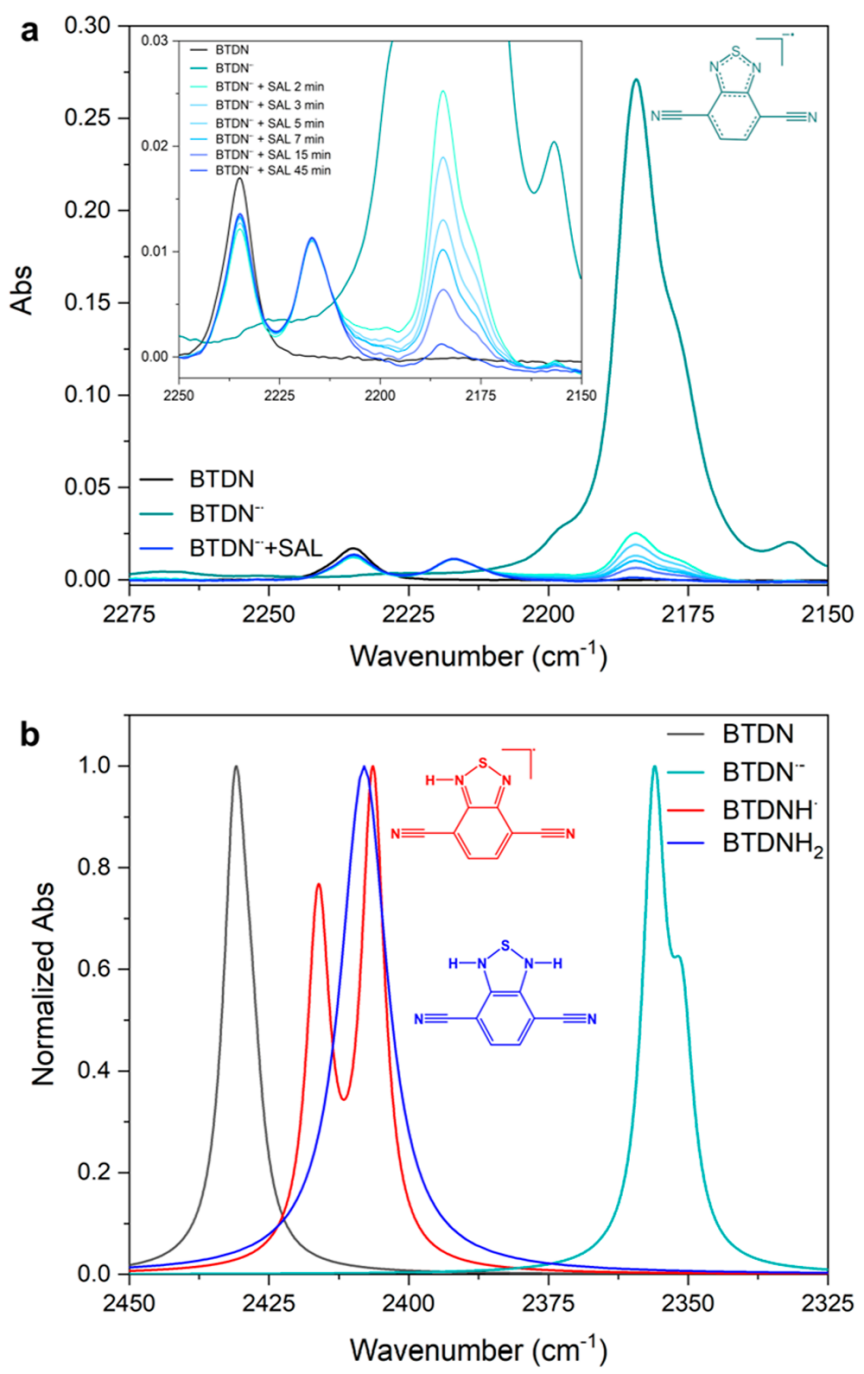
(a) Experimental and (b) calculated FTIR spectra of the catalytic intermediates, (a) BTDN (black), BTDN^–•^ (teal), BTDN^–•^ with the addition of 1 equiv. of SAL (BTDN^–•^ + SAL) (blue), and transitions in between the intermediates. Inset showing a zoom in on the decline in BTDN^–•^ and the growth of BTDNH_2_ species after the addition of 1 equiv. of SAL. (b) Calculated normalized spectra of BTDN (black), BTDN^–•^ (teal), BTDNH^•^ (red), and BTDNH_2_ (blue). Spectra extracted from DFT calculations at the M08-HX/6-311++G(d,p) theory level.

The new peak at 2217 cm^–1^ can be attributed to a new protonated species. Notably, half of the reactants return to the unperturbed BTDN species (Figure S17). Combining the electrochemical study with IR and the following DFT calculation, the new protonated species could be doubly protonated BTDN, BDTNH_2_,^25^ formed from monoprotonated organo-hydride of BTDN^–•^ followed by the DISP reaction between two BTDNH^•^ species: 2 BTDN^–•^ + 2H^+^ → 2BTDNH^•^ → BHDNH_2_ + BTDN. This reaction mechanism is further confirmed by running an operando FTIR-SEC experiment with a Pt working electrode at a potential of the first reduction (Figure S18). In this experiment all of BTDN can be observed first converting to BTDN^–•^, and then transforming to the new species with 2217 cm^–1^ peak, but without the 2235 cm^–1^ peak returning since the BTDN formed by the DISP reaction is reduced in situ again at the applied potential. The BTDNH^•^ intermediate that would be required for this step is not clearly visible in the IR spectra, the calculated IR of BTDNH^•^ with two split peaks shows a strong overlap with BDTNH_2_ (single peak, Figure 3b) which could explain the absence of BTDNH^•^ species in the spectra. It is also possible that DISP reaction is too fast to be observed under our experimental conditions.

Considering all the possible protonated species from BTDN^–•^, DFT calculations were applied. Comparing the free binding energy, Δ*G*, of protonation on the reduced BT at the N- or S-site it is clear that protonation on the N-site is by far the most likely scenario (Figure S21), because the protonation of S has much larger Δ*G* than that of the pronation of N. However, the intermediates of the final catalytic step were not captured experimentally due to reaction rates faster than the experimental detection limit. From transition state calculations, by far the most energetically favorable reaction seems to be a hydride donation reaction directly to a proton (SAL) in the solution from the reduced doubly protonated species BTDNH_2_^–^ (Figure S22).

Combining experimental and theoretical data, an electrocatalytic mechanism for hydrogen evolution from BTDN is therefore proposed, as shown in Figure 4. First, BTDN is reduced to BTDN^–•^ and followed by protonation of BTDN^–•^ radical anion at the N-site via an EC reaction in the presence of an acid to form an organic hydride radical BTDNH^•^. Subsequently, a net HAT reaction occurs between two BTDNH^•^ species to form the BTDNH_2_ species and BTDN. A more reductive voltage is needed to get into the final catalytic wave, where the BTDNH_2_ species is reduced again. And looking at the energetics of H_2_ formation, seemingly a hydride type transfer happens to form H_2_ and the BTDNH^•^ species which would react further. As no hydrogen was detected from the first reduction peak, it indicates that the second reduction of BTDNH_2_ is necessary for the catalytic process, further strengthening the proposed mechanism. However, participation of multiple protons in the final catalytic step with BTDNH_2_ as well as involvement of resonance structures of the intermediates during the catalysis could not be experimentally excluded and therefore needs to be further investigated.

**Figure 4:**
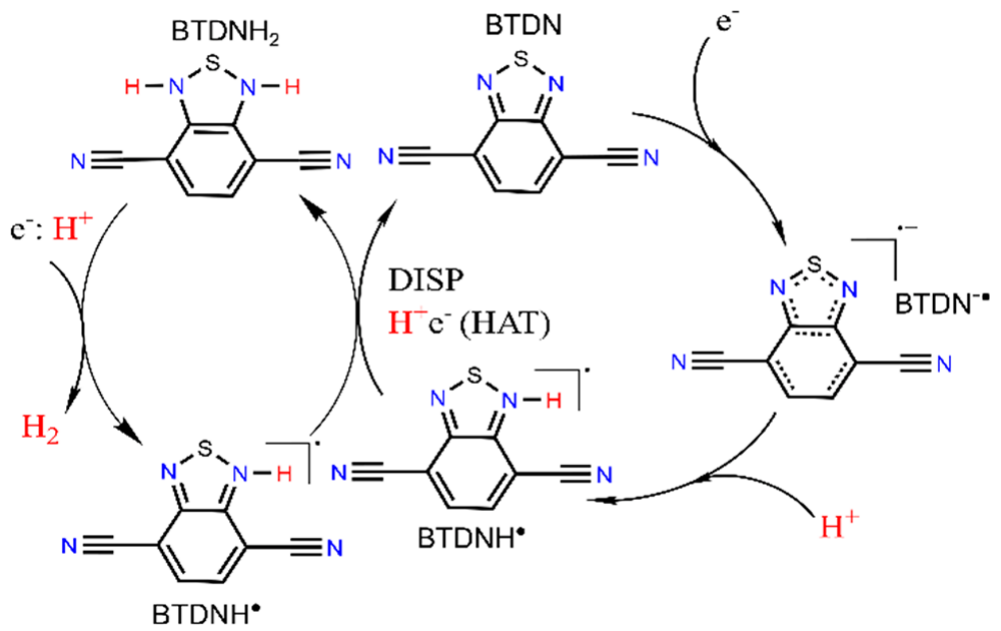
Proposed catalytic mechanism for electrocatalytic hydrogen evolution from BTDN.

In summary, we have demonstrated a small organic molecule 2,1,3-benzothiadiazole-4, 7-dicarbonitrile (BTDN) that shows electrocatalytic hydrogen evolution on glassy carbon electrode in the presence of salicylic acid in acetonitrile. Reaction intermediates have been captured or proposed according to EPR, UV–vis SEC, IR, NMR, and DFT calculation. Eventually, an electrocatalytic mechanism of BTDN for electrocatalytic hydrogen production is proposed. This work paves the road for development and study of small organic catalysts for hydrogen production. The result also provides information to understand the role of benzothiadiazole unit in some functional materials such as covalent organic framework and mesoporous organic semiconducting polymers used for electro- or photocatalytic hydrogen production. More research work on understanding the final catalytic step of BTDN for hydrogen production, investigating other potential reaction mechanism and studying the effect of BTDN-type molecular structures on catalytic hydrogen evolution, CO_2_ reduction, and organic photoredox catalysis is ongoing.

## Supporting Information Available

Experimental details, control experiments, calculation of TON, EPR spectrum, and determination of rate constant and the computational method/results (PDF)
