# Supplementary material for: Small Organic Molecule Based on Benzothiadiazole for Electrocatalytic Hydrogen Production
Source: J Am Chem Soc. 2021 Dec 2;143(50):21229–33. doi: 10.1021/jacs.1c10600 (PMC8704194; doi:10.1021/jacs.1c10600)
Supplement: Supplementary file 1 — ja1c10600_si_001.pdf [file ja1c10600_si_001.pdf]

Supporting Information

Small Organic Molecule Based on Benzothiadiazole for Electrocatalytic Hydrogen Production.

Martin Axelsson[1], Cleber F. N. Marchiori[2], Ping Huang[1], C. Moyses Araujo[2,3,*] and Haining Tian[1,*]

[1] Department of Chemistry-Ångström Laboratory, Uppsala University, SE 751 20 Uppsala, Sweden

[2] Department of Engineering and Physics, Karlstad University, 65188 Karlstad, Sweden

[3] Department of Physics and Astronomy, Ångström Laboratory, Uppsala University, 751 20 Uppsala, Sweden

* corresponding author: haining.tian@kemi.uu.se; moyses.araujo@kau.se

Experimental:

All other reagents were purchased from Sigma-Aldrich (Sweden) and used as received unless indicated otherwise. The potentiostat used for all electrochemical measurements was a AUTOLAB PGSTAT302N and a AUTOLAB PGSTAT302.

Cyclic voltammetry

The experiment was performed in a cylindrical ø 2.5 cm x 5 cm glass cell. The solvent used was spectroscopic grade acetonitrile (Uvasol, Sigma-Aldrich) dried over 3Å molecular sieves. A 3 mm diameter glassy carbon disc electrode was used as a working electrode which was polished carefully with 0.05-micron Al particle paste on a polishing pad (unless specified otherwise), a platinum wire was used as a counter electrode, and as a reference electrode a pseudo reference Ag/Ag^+ silver wire was used, covered with two vycor frits. As supporting electrolyte 0.15 M tetrabutylammonium hexafluorophosphate (TBA PF_6) was used (≥99.0 %, Sigma-Aldrich). Before use, TBAPF6 was recrystallised from ethanol and the dried in a vacuum oven at 85 ^oC.

The typical experiments were performed with 5 ml solvent with the supporting electrolyte was added to the cell and was then purged with argon. The voltammograms were recorded at varying scan rates (1 mVs^{-1} to 10 Vs^{-1}). To determine the potentials, ferrocene was added at the end of each experiment as an internal reference.

For a rough overpotential and to look at the feasibility of the SEC IR experiment voltammograms were also recorded with a 2 mm diameter Pt disc electrode as the working electrode.

Bulk electrolysis experiments

Bulk electrolysis was performed in two different cells. A H-cell made from two ø 21 mm x 61 mm cylinders connected by a ø 15 mm x 30 mm cylinder separated by a ø 15 mm x 2 mm porous glass frit. The other is the cylindrical ø 25 mm x 50 mm glass cell with a special rubber stopper and the counter electrode was also separated from the reaction solution in a small compartment covered with a vycor frit.

Radical generation

Glassy carbon foam connected with a glassy carbon rod was used as a working electrode. a Pt wire or a similar glassy carbon foam electrode was used as a counter electrode. As a reference electrode Ag/Ag^+ pseudo reference was used. The experiments were performed by first running a voltammetric scan to determine where the reduction potentials were. The potential is set at -1.1 V vs Ag/Ag^+ electrode for $BTDN^{\cdot-}$ generation. The experiment was then run with stirring until only background current remaining. Complete transformation into radical was confirmed by FTIR spectroscopy.

Electrocatalytic hydrogen evolution experiments

The working electrode was swapped out for a ø 5 mm x 100 mm glassy carbon rod to keep track of the exact surface area used. The counter electrode was the Pt wire and the reference electrode were Ag/Ag^+ reference electrode. Both the counter and reference electrodes were placed separate compartments allowing ion exchange through porous vycor frits and avoid any catalytic presence of the electrode materials[1] [1]. The solution used for the electrocatalytic experiment was 1 mM BTDN and 10 mM SAL. The control experiments were carried out without any substrate, but only with 10 mM SAL. The generated gas was then analysed and quantified with an HPR-20 gas analysis system (HIDEN Analytical) with Ar as a carrier gas.

UV-Vis spectro-electrochemistry (SEC) was performed in a 5 x 10 mm glass cuvette. A ca.1 mm thick piece of glassy carbon foam was used as the transparent working electrode. The Pt wire counter electrode and Ag wire pseudo reference system was used. A single fritted cell was used for the reference electrode. The spectra were recorded with a diode array spectrophotometer (Agilent 8453).

IR spectroscopy was measured using a Bruker Vertex 70V FTIR in transmission mode with a MCT detector. The cell was a flat cell with a 1 mm spacer and CaF windows, the solutions used where always based on a 5 mM BTDN solution and the species where then generated with bulk electrolysis and acid addition. FTIR SEC was measured with a Specac cell with a 1 mm spacer with inbuilt Pt-meshes as working electrode and counter electrode and a silver wire as pseudo reference electrode. A 5 mM BTDN solution in 0.2M TBA PF_6 THF was used for the measurement.

Steady state UV-Vis spectroscopy was performed with a Varian Cary 5000 spectrophotometer. All spectra where taken in a 1x1 cm quartz cuvette in transmission mode and the solutions where diluted to have a max absorbance of 0.5.

NMR was measured in d_3-AcN with a Jeol Resonance 400 MHz spectrometer at 293 K.

EPR All EPR spectra were recorded on a Bruker EMX-micro spectrometer equipped with an EMX-Primium bridge and an ER4119HS resonator. The settings were; Microwave frequency 9.86 GHz, power, 1.2 mW; Modulation frequency 100 kHz, amplitude 0.02 mT and the spectra were recorded under ambient condition.

Voltammetry

Three different organic acids have been applied for this experiment. Trifluoroacetic acid (TFA), Acetic acid (AcA) and Salicylic acid (SAL), all are proven to be appropriate proton donors for studying hydrogen evolution in AcN[2]. A similar perturbation of the CV occurs with all of the acids but with different magnitudes depending on the pK_a of the acid (Figure S1). TFA being

the strongest acid with a pK_a of 12.7 in AcN, shows the largest positive shift for the first reduction (Figure S3)[3], implying that the reduced species reacts the fastest with the strongest acid as expected. TFA has an issue of a very small window for catalysis before TFA is catalysed by glassy carbon (Figure S4). The weak acid AcA with a pK_a of 23.51 in AcN[4] cannot fully protonate the $BTDN^{-\cdot}$ species during CV scan, as the presence of the oxidation peak of the BTDN/ $BTDN^{-\cdot}$ couple is obviously observed. (Figure S5). When using SAL, only a small amount of oxidative current is returned from the first reduction, the back current is expected to be from oxidation of $BTDN^{-\bullet}$ that has yet to react with acid during this time frame.

At high acid concentrations (≥ 8 mM) a curve crossing behavior has been observed in the catalytic wave at slower scan rates (10 $mV{\cdot}s^{-1}$–200 $mV{\cdot}s^{-1}$, Figure S6). Curve crossing means that the catalytic current is higher on the return scan which is often indicative of build-up of the catalytic species during the scan. The curve crossing is attributed to two different processes, non-catalytic degradation of SAL, and the buildup of catalytically relevant intermediates. After cycling in SAL, the onset potential for the reductive wave moves to less reductive potentials (Figure S7) seemingly, due to the formation of a layer on the working electrode, this current does not seem to be catalytic since very little hydrogen is detected from the bulk electrolysis experiment (Figure 1b). The second process would be due to the complex reaction kinetics in the formation of catalytic intermediates which would mean that the reaction is not yet at equilibrium or the DISP process is not complete at the beginning of the catalytic wave.

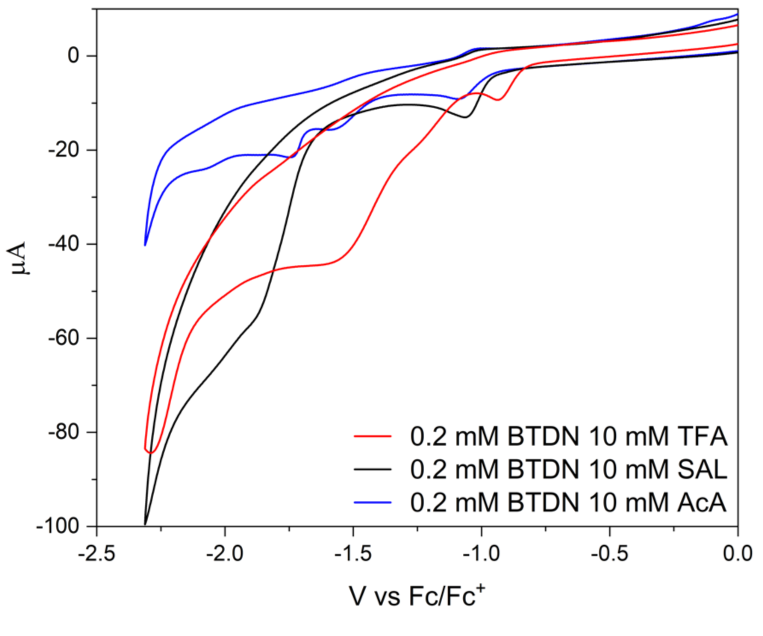

Figure S1. Comparison of CVs of BTDN with the three organic acids TFA (red), AcA (blue) and SAL (black).

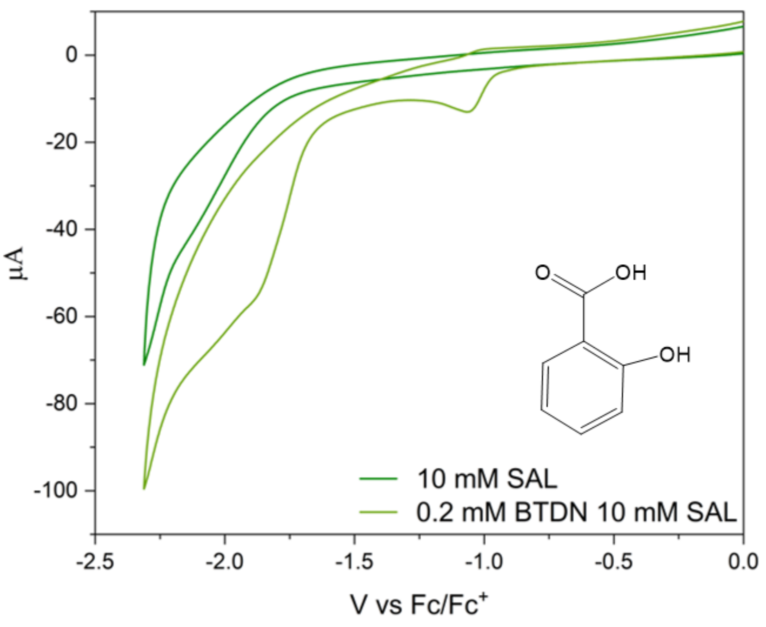

Figure S2. CVs of 10 mM SAL on a glassy carbon electrode, with BTDN (olive) and without BTDN (green).

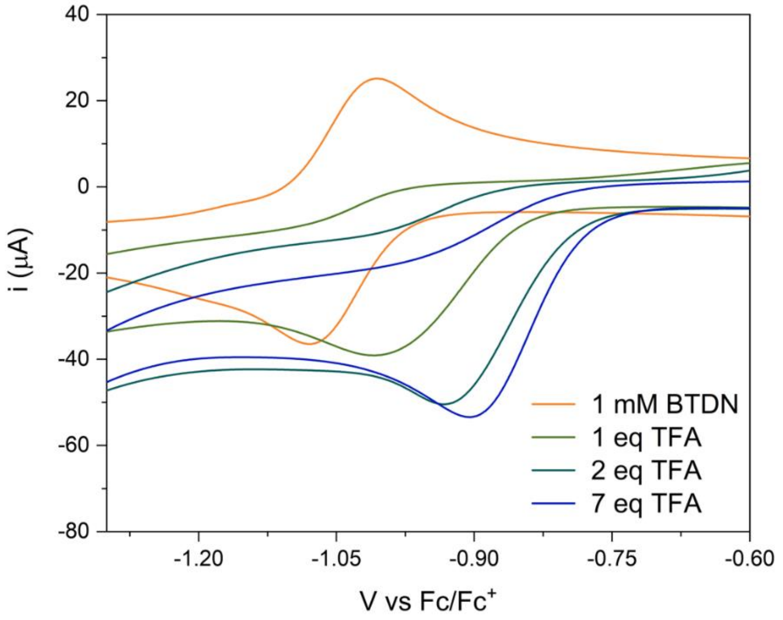

Figure S3. Showing the positive shift of the reduction as more TFA is added to a BTDN solution, more than 100 mV at 7 equivalents of acid.

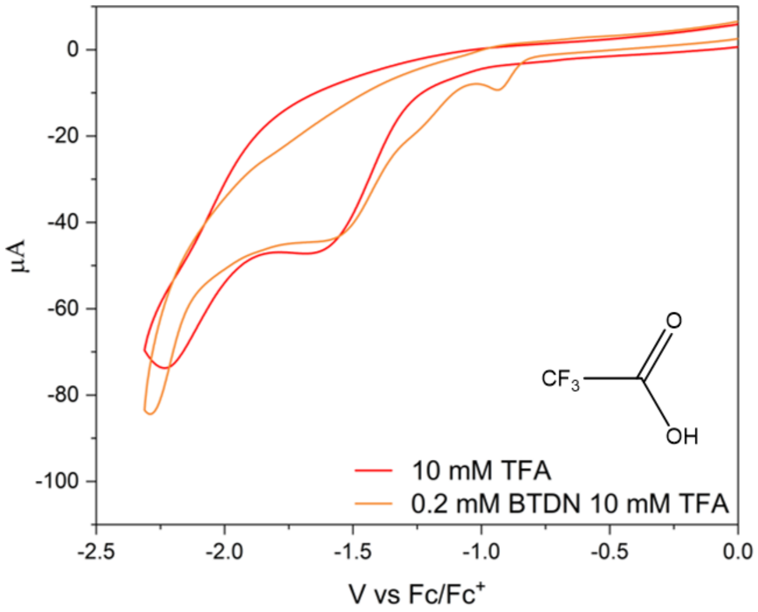

Figure S4. CVs of 10 mM TFA on a glassy carbon electrode, with BTDN (orange) and without BTDN (red).

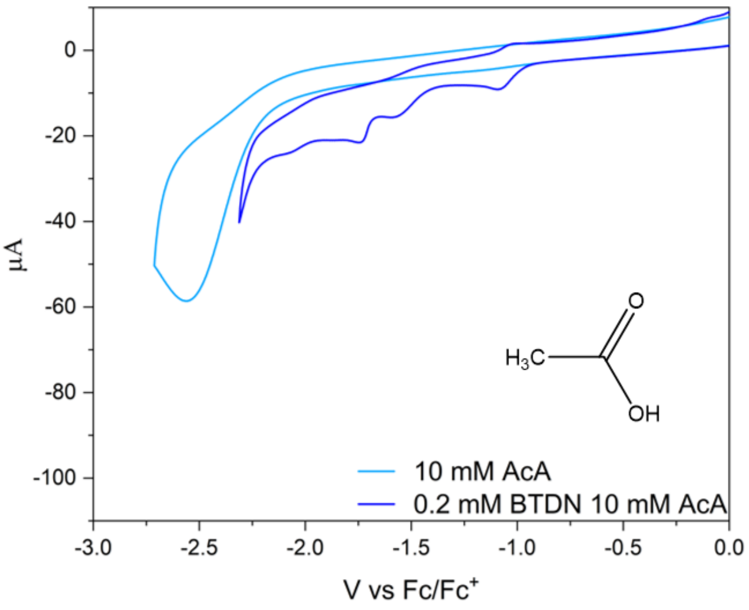

Figure S5. CVs of 10 mM AcA on a glassy carbon electrode, with BTDN (blue) and without BTDN (light blue).

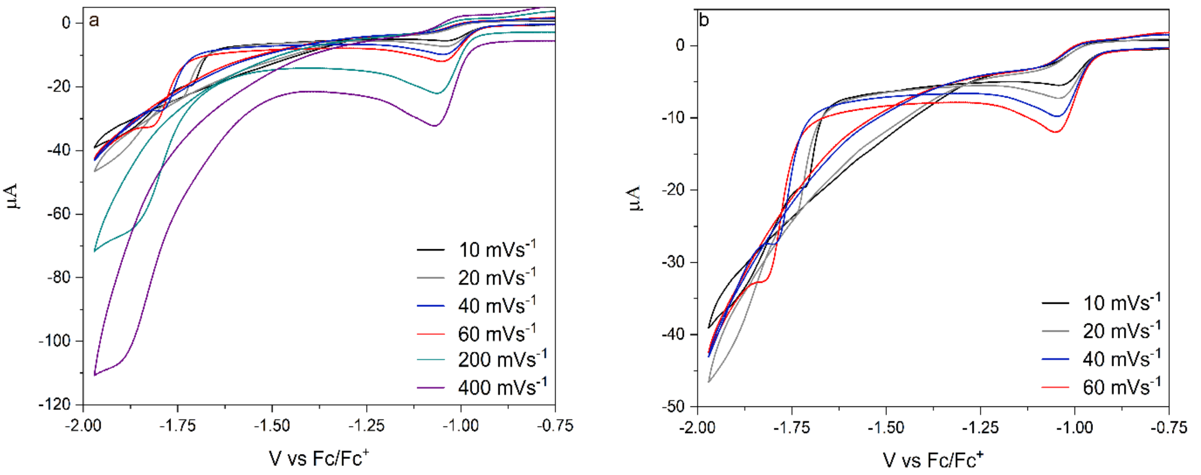

Figure S6. Scan rate dependence of 0.2 mM BTDN and 10 mM SAL showing curve-crossing behaviour from 10 mVs^{-1} to 200 mVs^{-1}. (a) All of the scan rates (b) zoom in on the slower scan rates

Rinse tests shows the formation of a layer of decomposed SAL on the surface of the glassy carbon (Figure S7). The layer is not redox active but shifts the reduction peak of SAL which also shifts the CV when BTDN is present, however since very little H_2 is detected from just SAL (Figure 2 b)) the SAL reduction seems to be mostly non-catalytic. After a few equivalents of acid is added to the solution with BTDN the catalytic current increases mostly linearly at multiple scan rates (Figure S8).

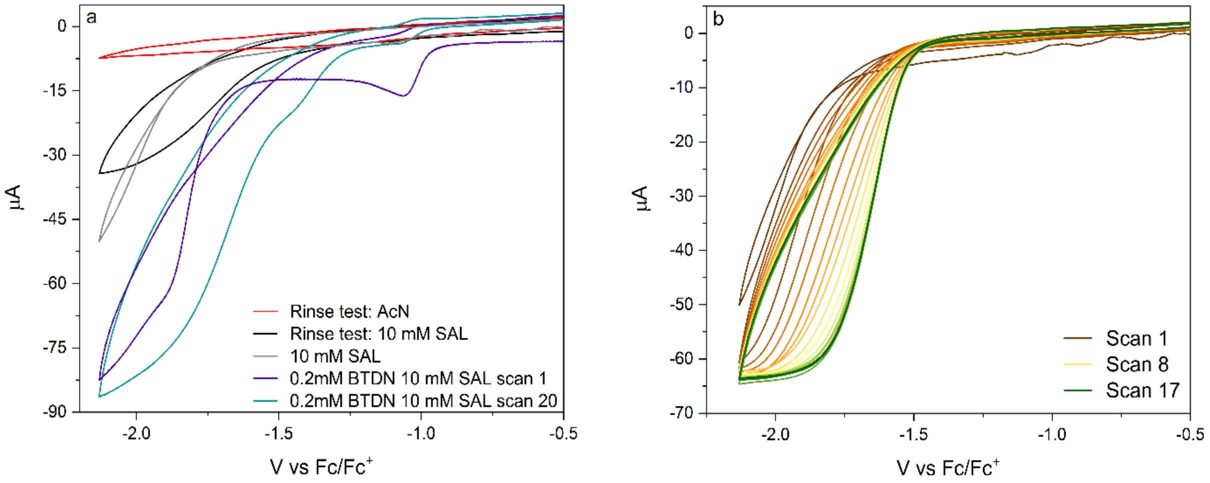

Figure S7. Behaviour after multiple scans in BTDN + SAL and prestine SAL a) Rinse test in can and 10 mM SAL after 20 scans in a 0.2 mM BTDN 10 mM SAL solution, shows behaviour in just AcN (Red) and in 10 mM SAL. b) Shift of SAL reduction over multiple sequential scans.

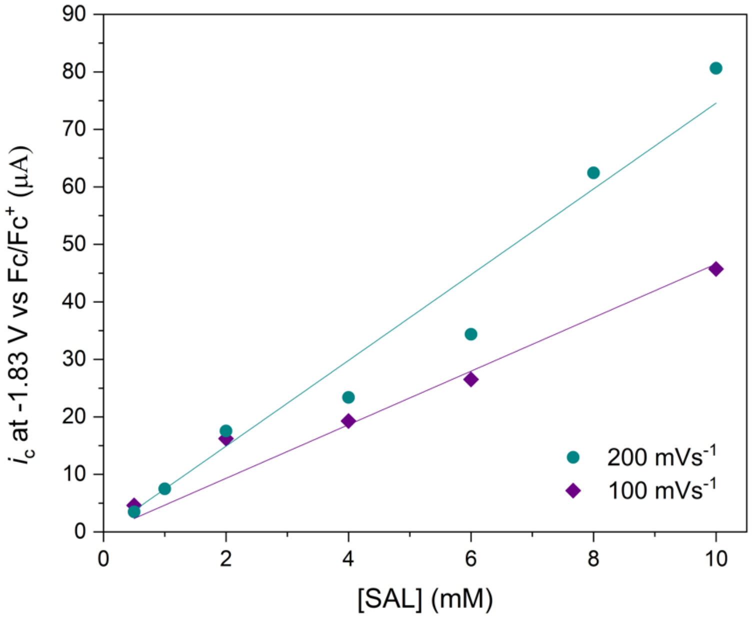

Figure S8. The catalytic current at -1.83 V vs Fc/Fc$^+$, increasing as a linear function of the added concentration of SAL at 200 mVs^{-1} (Teal) and 100 mVs^{-1} (Purple).

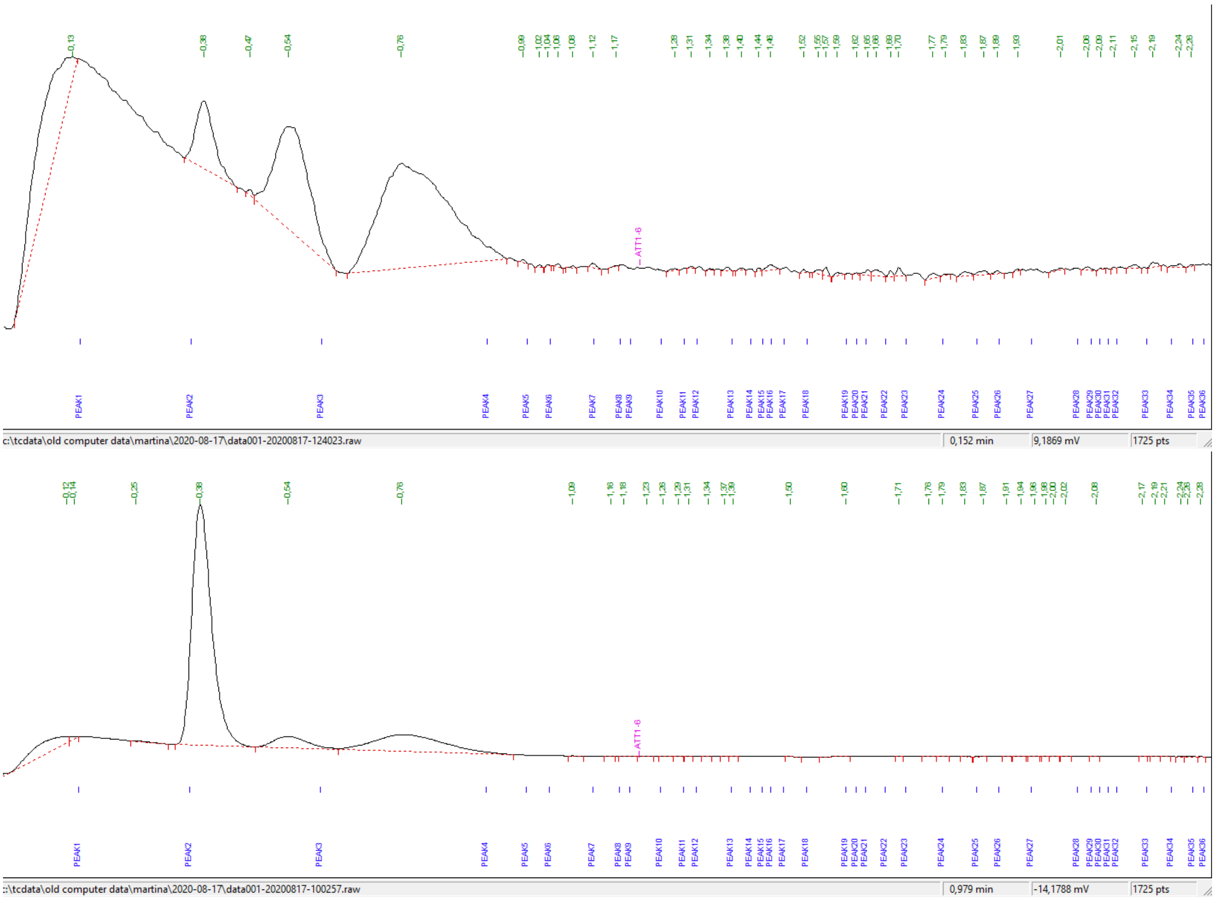

Figure S9: GC data of H_2 evolution from only SAL (Upper) and BTDN + SAL (Lower), the first peak represents H_2 and the background is comparable on both measurements.

Mechanistic analysis

Mechanistic analysis of the reduction was performed by studying the value of the potential at the peak current of the reduction, the peak potential. The peak potential is plotted versus the decimal logarithm of the scan rate, and the concentration of BTDN, as they are varied in the system. The slope attained is then characteristic for the Nernstian description for the total mechanism of the peak[4-6].

Initially, the increase in current of the reduction peak when an acid is introduced to the system, means that more electrons are transferred (Figure 1), this increase in current would fit well with either an ECE (electrochemical-chemical-electrochemical) type mechanism or to a disproportionation (DISP) type mechanism[4]. However, at lower concentrations of BTDN (50 µM) the current does not increase (Figure S9). The change with concentration indicates that the mechanism at higher concentrations, is dependent on the concentration of a species formed in the reaction. This type of mechanism corresponds well to a variation of a DISP mechanism, where electron transfer takes place in the homogeneously in the solution and not at the electrode surface. Comparatively an ECE mechanism would not be expected to be concentration dependent.

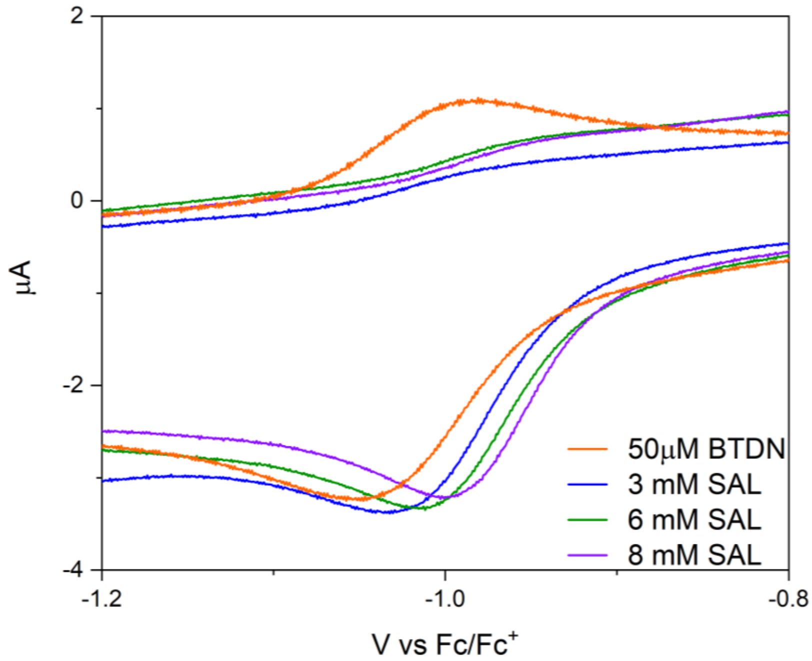

Figure S10. The first reduction of BTDN when titrated with SAL, showing no or very low increase in current.

The concentration dependence of the peak potential is close to 0 mV for both mechanisms (Figure S10), with a slight shift on peak potential. And the scan rate dependence for both mechanisms showing a dependence close to -30 mV which would be expected for both mechanisms, excluding a DISP 2 type mechanism.

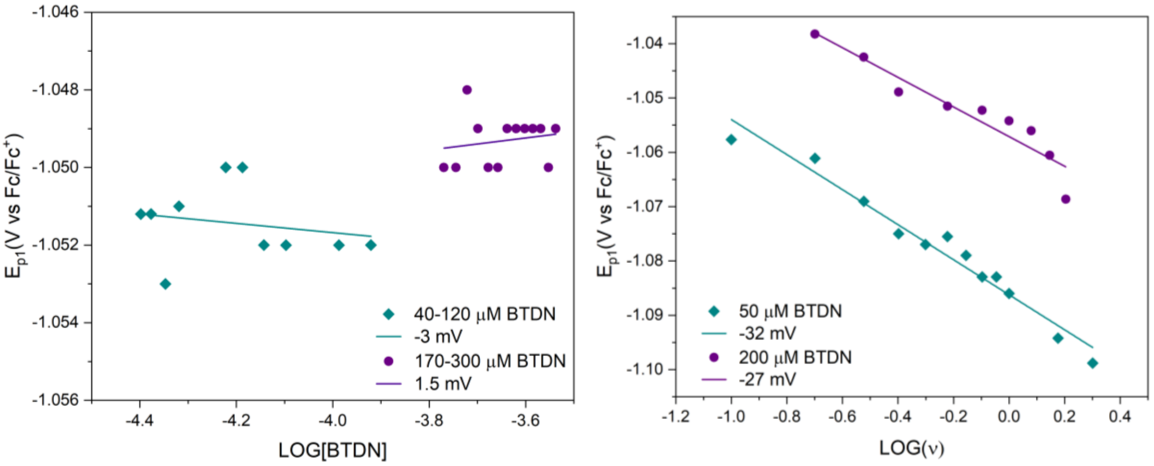

Figure S11. The peak potential dependence on the logarithm a) concentration of BTDN in the ranges of 40 - 120 µM and 170 – 300 µM with slopes close to 0 mV, and b) scan rate at 50 µM (100 mVs^{-1} – 4 Vs^{-1}) and 200 µM (200 mVs^{-1} – 1.6 Vs^{-1}) with slopes close to –30 mV. All measurements including 10 mM SAL.

The higher concentration mechanism does however, not seem like the standard type described in literature, from the FTIR experiments where SAL and BTDN$^{-\bullet}$ react the apparent formation of $BTDNH_2$ takes place. This indicates that both not just an electron but a proton is transferred as well after the first chemical step, this seemingly would go from an N-H bond to form another, similar bond, and would therefore occur as a hydrogen atom transfer (HAT) (Scheme S1).

The HAT mechanism seems like the only plausible option since this reaction only occurs at concentrations when two BTDNH$^\bullet$ species could interact in the duration of a scan. That means that the second reduction cannot happen first, since an increase in current would be visible even in lower concentrations. If the protonation would happen first, this should also happen at the lower concentration of BTDN since there is an abundance of SAL in the solution. A concerted HAT process from one BTDNH$^\bullet$ species to another therefore seems like the only option.

$$\mathrm{BTDN} + e^- \rightleftharpoons \mathrm{BTDN}^{-\bullet}$$

$$\mathrm{BTDN}^{-\bullet} + \mathrm{H}^+ \longrightarrow \mathrm{BTDNH}^\bullet$$

$$2\ \mathrm{BTDNH}^\bullet \longrightarrow \mathrm{BTDNH_2} + \mathrm{BTDN}$$

Scheme S1. The proposed mechanism following the first reduction of BTDN.

Simulations

To confirm that the proposed mechanism would fit the CV, the data is simulated with DigiElch 8.0. Diffusion and the initial electron transfer kinetics from the electrode (*ks*) parameters are extracted by fitting the unperturbed BTDN CV, giving values of $D_{BTDN} = 4*10^{-6}$ cm^2 s^{-1} which is assumed for all other BTDN based species, and a value of *ks* = 0.02 cm s^{-1}. With these values the first reduction can be fit with the initial protonation at a rate of $6*10^3$ s^{-1} and the HAT at a rate $9*10^3$ s^{-1} M^{-1}, these parameters fit experimental data well over a variety of scan rates (Figure S11). To properly evaluate these kinetics, a more careful study would

have to be made using transient spectroscopy.

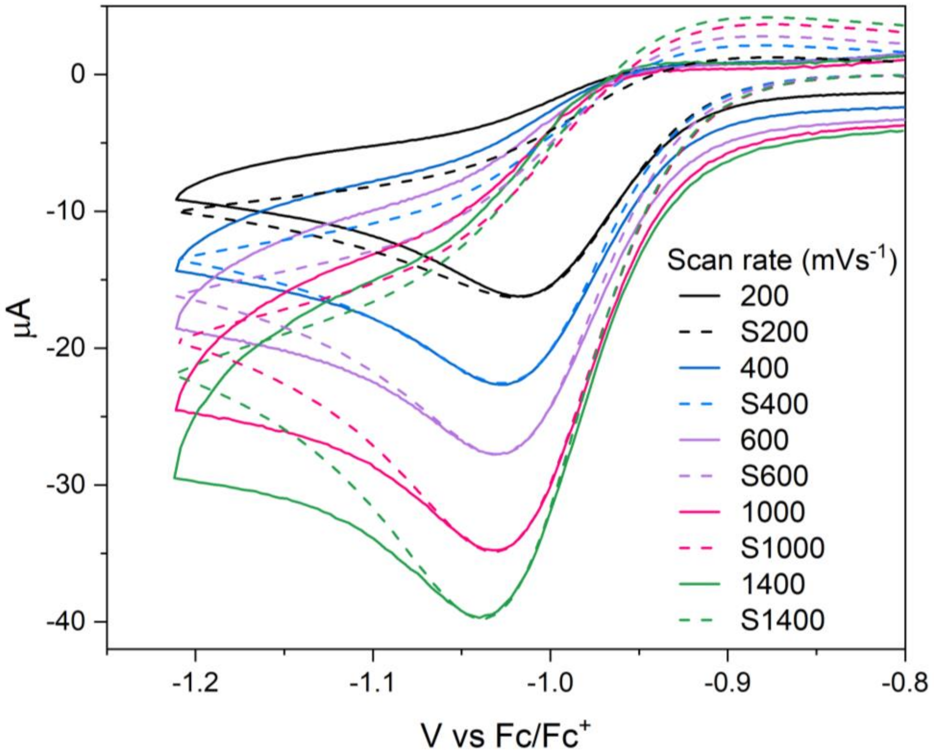

Figure S12. Simulated CVs (dashed) of 0.2 mM BTDN with 10 mM H^+ compared to experimental data (full).

Faradaic efficiency and TON calculation

Faradaic efficiency is calculated as (3) where n_{H_2} is the moles of H_2 generated, n_{e^-} is the number of electrons required for one H_2 in this case 2, F is faradays constant and Q is the total current from the experiment.

$$f = \frac{n_{H_2} n_{e^-} F}{Q} \qquad (3)$$

The amount of hydrogen gas that is generated measured by injecting 100 µl gas in to the GC and the H_2 is quantified from a calibration curve. The total amount of H_2 is calculated from the H_2 in the headspace of the cell by using Henry's law. The Henry's law volatility constant $K_H^{Px} \approx 5.7 * 10^8$ Pa derived from data about solubility of H_2 in AcN at different partial pressures at 298,15 K[6]. TON is calculated as (4), dividing the total amount of evolved H_2 with the total amount of BTDN in the solution.

$$TON = \frac{n_{H_2}}{n_{BTDN}} \qquad (4)$$

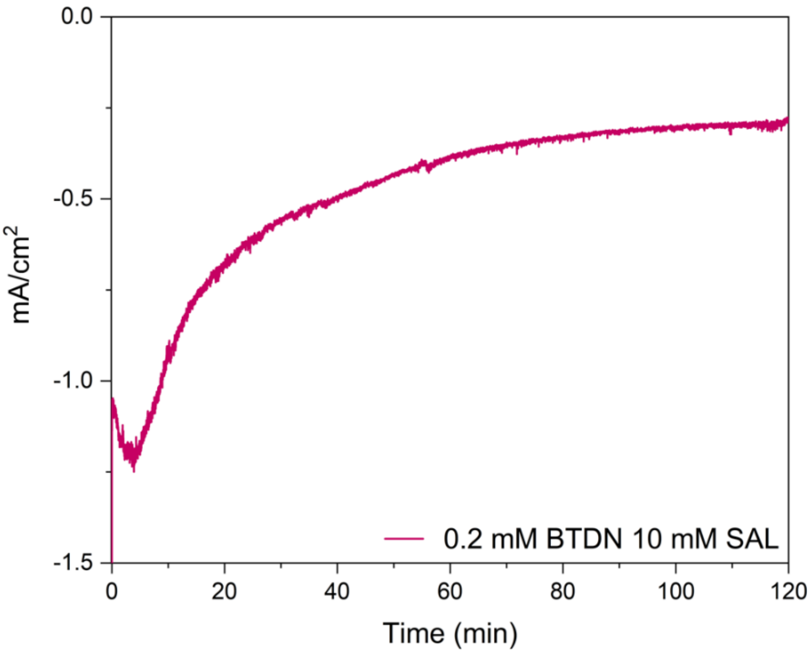

Figure S13. TON experiment, bulk electrolysis run for 2h at -1.65 V vs Fc/Fc^+ with 0.2 mM BTDN and 10 mM SAL.

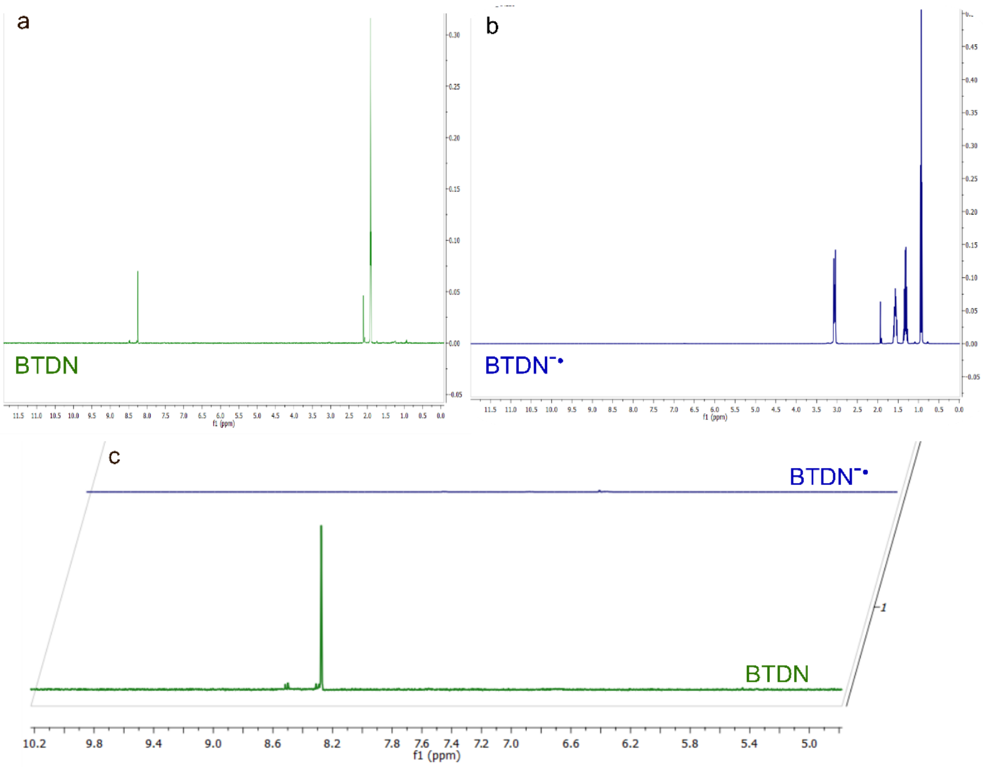

Figure S14. NMR of BTDN (*g*reen a) in AcN-d3, $BTDN^{\cdot-}$ (*b*lue b) in 0.15 mM TBA-PF_6 AcN-d3 in 0.15 mM TBA-PF_6 AcN-d3. c) The aromatic region of both species compared.

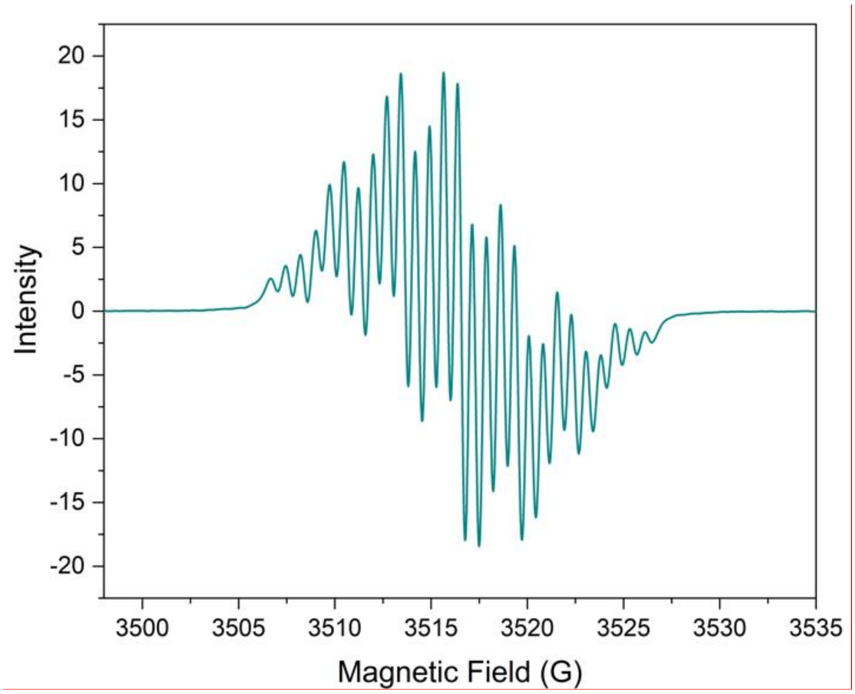

Figure S9. EPR spectrum of the BTDN$^{\cdot -}$ radical anion.

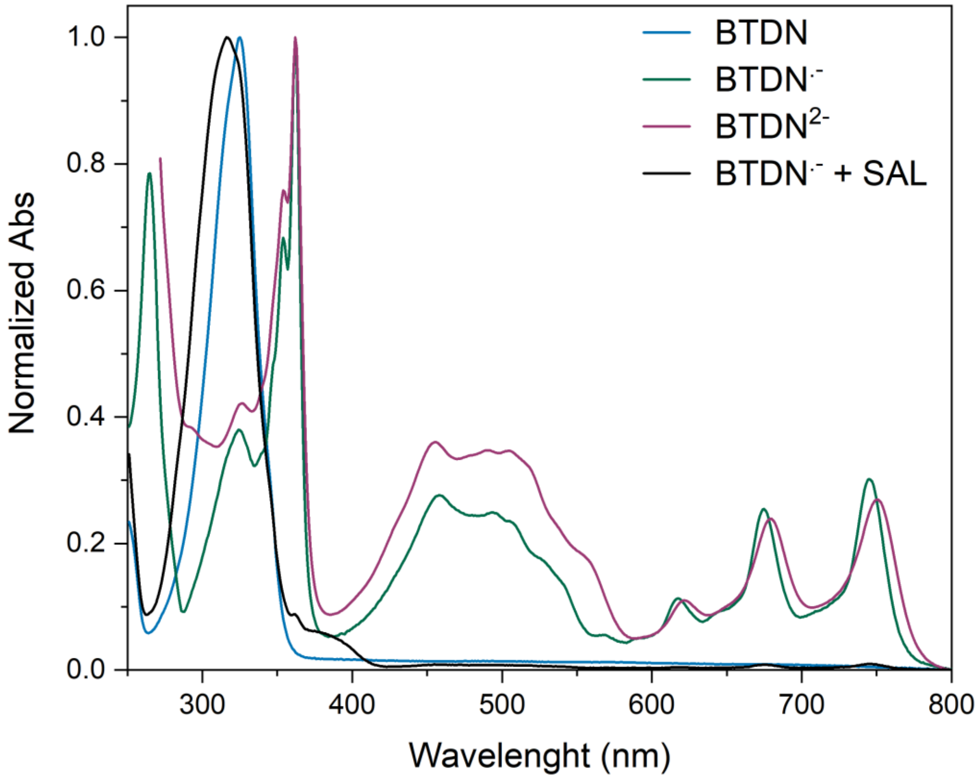

Figure S10. Steady state UV-Vis of BTDN (Blue), BTDN$^{\cdot -}$ (Green), BTDN^{2-} (Purple), BTDN$^{\cdot -}$+SAL (Black).

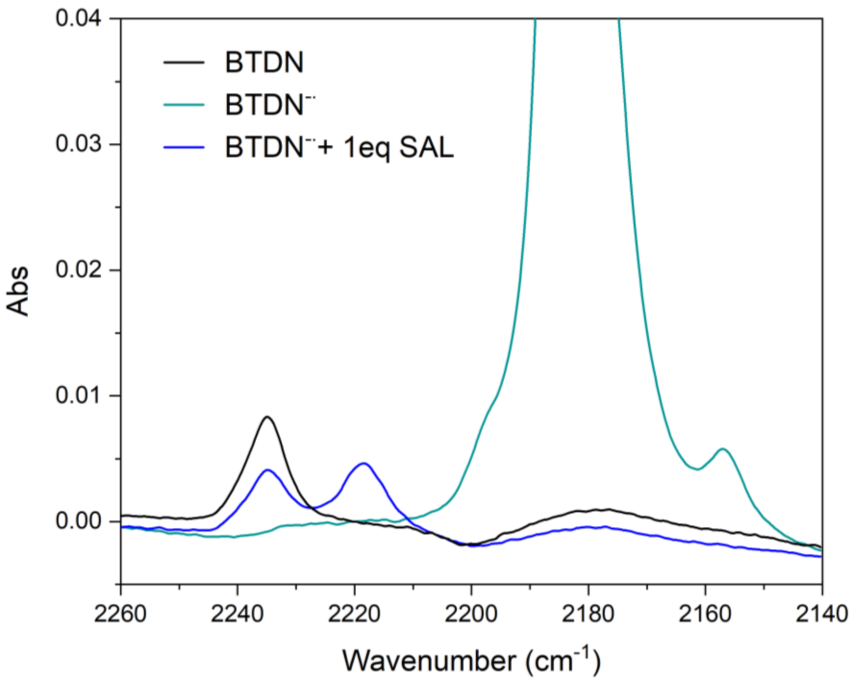

Figure S17. FTIR of BTDN, $BTDN^{-\bullet}$ and $BTDN^{-\bullet}$ in the presence of 1 eq SAL, showing that the the BTDN peak (2235 cm^{-1}) has been reduced to half as the $BTDNH_2$ peak (2217 cm^{-1}) rises, when SAL is added to $BTDN^{-\bullet}$.

Voltammetry with platinum electrodes

To investigate if operando SEC-FTIR could be performed to investigate the build up of reduced BTDN intermediates, CVs where measured with Pt disk electrode (Figure S15). By assuming no overpotential reduction of SAL on PT, these measurements we can get a rough estimate of the overpotential of the catalytic HER with BTDN and SAL. If looking at the half wave potential the overpotential would be about 500 mV and if looking at peak potential the overpotential would be about 300 mV. The BTDN reduction peak is also visible and almost identical to when the reduction occurs at a GC electrode, with this result the operando SEC-FTIR experiment was cunducted at about -1.3 V vs Fc/Fc^{+}(uncetrenty from the unreliable reference electrode in the SEC-FTIR cell).

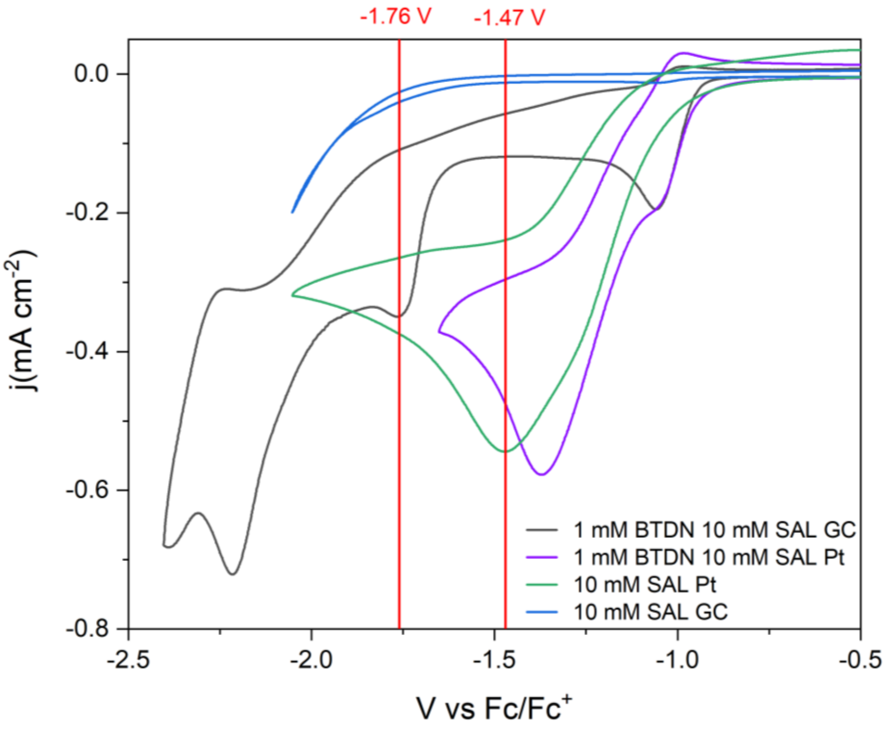

Figure S18. Comparing the reduction of SAL on GC and Pt electrodes, both with the clean electode on GC (blue) and Pt (green), as well as in the presence of 1 mM BTDN with GC (gray) and Pt (purple). Lines indicating the peak of the catalytic reduction on Pt and on GC in the presence of BTDN.

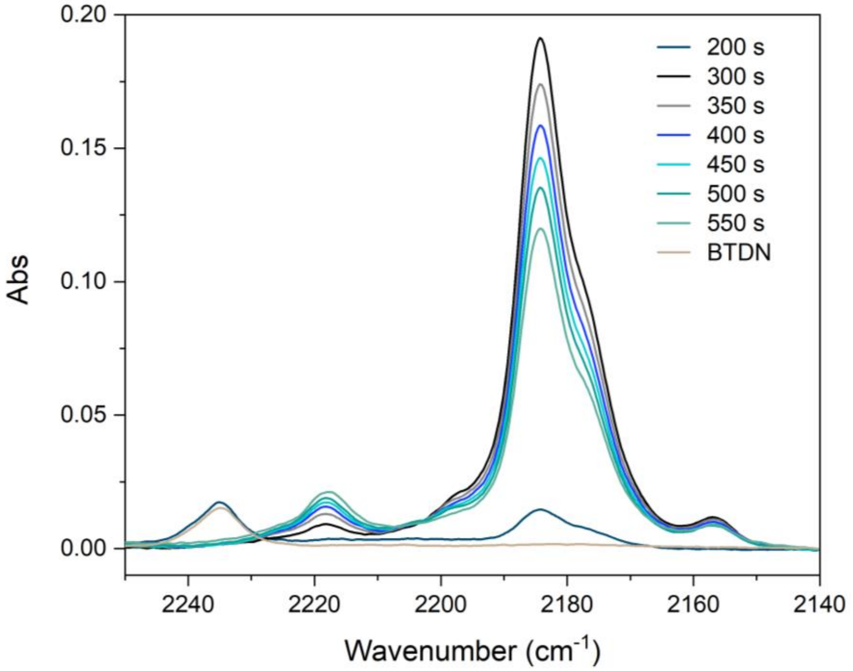

Figure S19. SEC FTIR of BTDN in the presence of 4 eq SAL, showing the disappearance of the BTDN peak (2235 cm^{-1}) and the rise and fall of the $BTDN^{-•}$ peak (2183 cm^{-1}) as well as the rise of the $BTDNH_2$ peak (2217 cm^{-1}).

Proton NMR of BTDN and $BTDN^{-•}$ were measured (Figure S19). Normal BTDN shows a single peak in aromatic region. When BTDN is reduced to $BTDN^{-•}$, the aromatic peak is vanished during that region because of the paramagnetic nature of the $BTDN^{-•}$ radical.

Computational procedure

The atomic scale modeling were conducted within the Density Functional Theory (DFT) framework employing M08-HX[9] as exchange-correlation functional and 6-311G++(d,p) as basis set [10–14] using the SMD continuum model [11] for solvation having acetonitrile as solvent. The IR spectra were obtained directly from frequency calculation. All calculations were performed using Gaussian16 package.

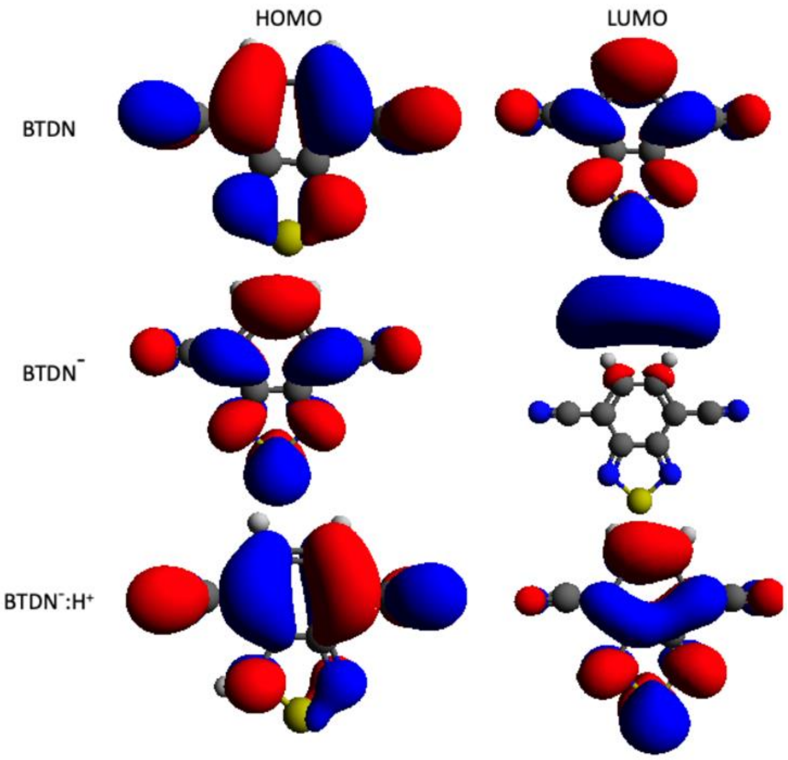

Figure S11 Spatial distribution of the near-gap molecular orbitals for the pristine molecule (BTDN) reduced state/radical (BTDN^{-}), reduced state/radical protonated (BTDN^{-}:H^{+}) and the reduced/radical protonated after the second reduction process (BTDN^{-}:H^{+})$^{-}$. Orbital obtained from DFT calculation at M08-HX/6-311++G(d,p) theory level (isovalue= 0.02).

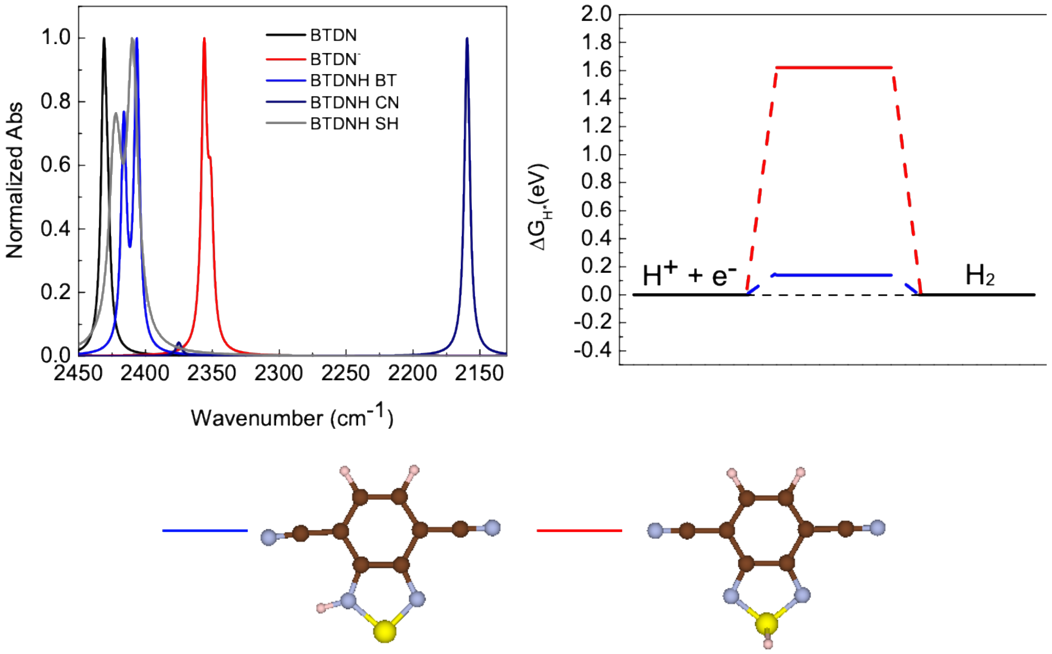

Figure S12 Left: Calculated IR spectra for the pristine molecule (BTDN) reduced state/radical (BTDN$^-$) and for the protonated reduced state/radical considering three possible sites for protonation: at the N atom from the BT unit (BTDNH BT), at the N atom from the CN group (BTDNH CN) and at the S atom (BTDNH SH). Spectra extracted from DFT calculation at M08-HX/6-311++G(d,p) theory level. Right: Calculated Hydrogen biding free energy obtained for two different pronation sites: N atom at BT unit and S atom.

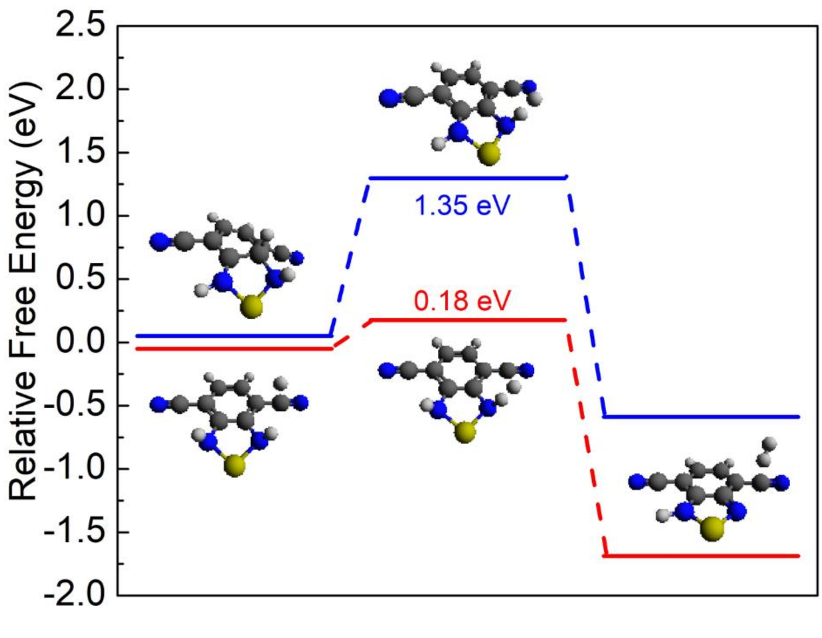

Figure S22. Calculated energy barriers for H_2 formation from interacting hydrogen atoms in a BTDN-H_3 model structures. The free energies values are referenced to the ground state geometries. The insets depict the structure of the reactants, transition states and the products. The barriers were obtained from DFT calculation at M08-HX/6-311++G(d,p) theory level. The transition states were obtained from DFT calculation employing the synchronous transit-guided quasi-Newton approach (QST3) to optimize the transition state.

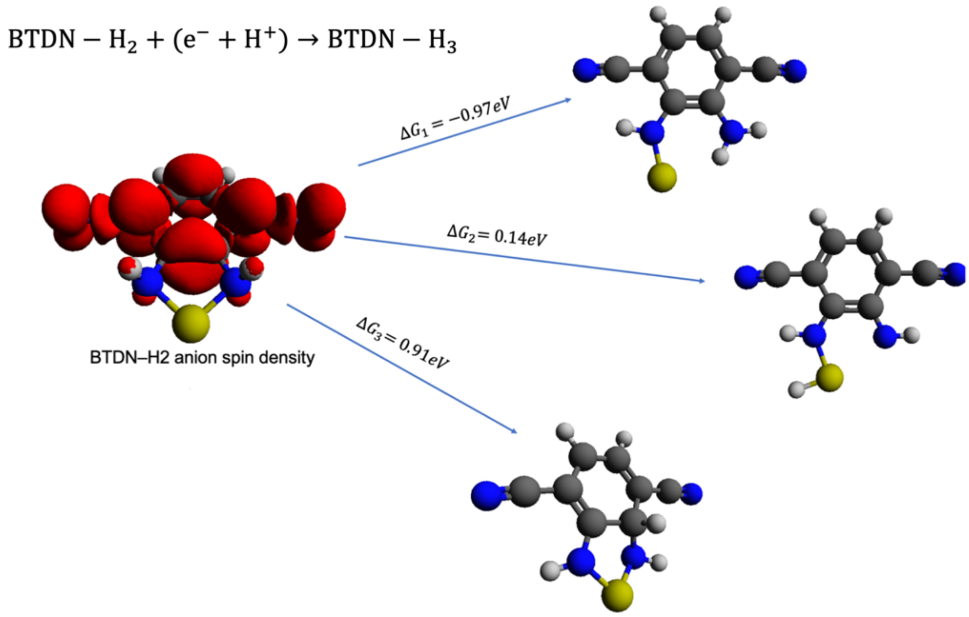

Figure S23. Schematic diagram illustrating the Gibbs free energy to the formation of BTDN-H_3 species along with the spin density of BTDN-H_2 anion. The Gibbs free energy variation for these reactions were obtained from DFT calculations at M08-HX/6-311++G(d,p) theory level.

References

(1) Costentin, C.; Savéant, J.-M.; Tard, C. *ACS Energy Lett.*, **2018**, 3, 695−703.

(2) McCarthy, B. D.; Martin, D. J.; Rountree, E. S.; Ullman A. C.; Dempsey, J. L. *Inorg. Chem.*, **2014**, 53, 8350−8361.

(3) Eckert, F., Leito, I.; Kaljurand, I.; Kütt, A.; Klamt, A.; Diedenhofen, M. *J. Comp. Chem.*, **2009**, 30, 799-810.

(4) Kütt, A.; Leito, I.; Kaljurand, I.; Sooväli, L.; Vlasov, V. M.; Yagupolskii, L. M.; Koppel, l. A. *J. Org. Chem.*, **2006**, 71, 2829-2838.

(5) Savéant, J.-M. Elements of Molecular and Biomolecular Electro-chemistry: An Electrochemical Approach to Electron Transfer Chemistry; John Wiley & Sons, 2006.

(6) Andrieux, C.P.; Hapiot, P.; Savéant, J.-M. Repetitive cyclic voltammetry of irreversible systems, *J. Electroanal. Chem.*, 349, **1993**, 299-309

(7) Amatore, C.; Gareil M.; Savéant, J.-M., Homogeneous vs. Heterogeneous electron transfer in Electrochemical reactions Application to the electro hydrogenation of anthracene and related reactions, *J. Electroanal. Chem.*, 147, **1983**, 1-38

(8) Brunner, E.; *J. Chem. Eng. Data*, **1985**, 30, 269–273.

(9) Zhao, Y.; Truhlar, D. G. *J. Chem. Theory Comput.*, , DOI:10.1021/ct800246v.

(10) McLean, A. D.; Chandler, G. S. *J. Chem. Phys.*, **1980**, 72, 5639–5648.

(11) Krishnan, R.; Binkley, J. S.; Seeger R.; Pople, J. A. *J. Chem. Phys.*, **1980**,72, 650-654.

(12) Frisch, M. J.; Pople, J. A.; Binkley, J. S. *J. Chem. Phys.*, **1984**, 80, 3265–3269.

(13) Marenich, A. V.; Cramer, C. J.; Truhlar, D. G. *J. Phys. Chem. B*, **2009**, 113, 6378–6396.
